# Efficient p–n Heterojunction Construction in Co_3_O_4_/Zn_0.4_Cd_0.6_S Composite for Boosted Visible-Light Photocatalytic H_2_ Production

**DOI:** 10.3390/nano16211397

**Published:** 2026-10-31

**Authors:** Yanzhu Ye, Zhongshui Li, Alian Wang, Shen Lin

**Affiliations:** 1 Training Management Division, Fujian Institute of Education, Fuzhou 350001, China; yanzhuye@126.com (Y.Y.); wal0922@163.com (A.W.); 2 College of Chemistry and Materials Science, Fujian Normal University, Fuzhou 350007, China; zsli@fjnu.edu.cn

**Keywords:** p–n heterojunction, Co_3_O_4_, Zn_0.4_Cd_0.6_S, photocatalytic H_2_ evolution, water splitting

## Abstract

In photocatalyst design, constructing p–n heterojunctions is a highly effective way to suppress photogenerated charge recombination and enhance photocatalytic activity. In this study, a precious-metal-free Co_3_O_4_/Zn_0.4_Cd_0.6_S p–n heterojunction composite was prepared using a facile solvothermal approach. The photocatalytic activity and long-term stability are significantly promoted by the introduction of Co_3_O_4_. The composite containing 4 wt% Co_3_O_4_ demonstrates a maximum H_2_ generation yield of 56.63 mmol g^−1^ h^−1^, which represents a 6.8-fold improvement over pristine Zn_0.4_Cd_0.6_S, significantly surpassing many comparable photocatalysts and even exceeding that of some noble-metal-modified composites. The apparent quantum efficiency (AQE) of 4 wt%-Co_3_O_4_/Zn_0.4_Cd_0.6_S reaches up to 21.86% at 420 nm. Systematic characterization reveals that the photocatalysts’ p–n heterojunction underlies the remarkable improvement in activity and durability of the composite through facilitated separation and migration of photoinduced electrons and holes. The p–n heterojunction-driven charge separation enhancement fundamentally underpins this significant performance leap. These findings demonstrate a promising approach for developing cost-effective, high-activity photocatalysts for sustainable energy applications.

## 1. Introduction

Growing concerns over intensifying energy shortages and environmental pollution are driving global research efforts toward new and renewable energy development [1,2,3,4,5,6]. Among various energy resources, hydrogen emerges as the frontier energy carrier owing to three inherent advantages: zero-emission potential, biological safety, and high enthalpy liberation [7,8,9,10,11,12,13,14,15,16]. The widespread application of hydrogen energy depends crucially on developing an efficient strategy for hydrogen production that does not rely on fossil fuels. The solar-powered photocatalytic water-splitting process demonstrates significant potential as a sustainable hydrogen generation strategy. Nevertheless, conventional photocatalysts face multiple limitations—including swift recombination of photoinduced charges, narrow-band UV activation, and poor corrosion resistance—constraining their practical deployment [17,18,19,20,21,22]. Designing and developing visible-light-responsive photocatalysts with excellent catalytic performance is essential for maximizing solar energy utilization.

In recent years, numerous semiconductor-based photocatalytic materials have been explored to develop visible-light-active photocatalysts to further the hydrogen evolution. Zn_x_Cd_1−x_S solid solutions, with their tunable optical properties and strong visible-light response, have emerged as particularly promising materials. However, standalone application of Zn_x_Cd_1−x_S still encounters many challenges, including low catalytic activity, fast charge recombination, and photocorrosion issues. To address these limitations, multiple strategies have been developed to suppress photogenerated charge recombination and improve photocatalytic hydrogen evolution performance. These include constructing heterojunctions, introducing ion doping, and modifying with co-catalysts, all of which have demonstrated effective improvements in charge separation efficiency and catalytic activity [23,24,25,26,27]. Cobalt-based compounds (e.g., CoO, Co_3_O_4_, CoS, CoP, Co(OH)_2_), distinguished by their unique structural features and excellent catalytic properties, have been widely employed in photocatalysis [28,29,30,31,32]. In particular, Co_3_O_4_ has garnered substantial research interest owing to its excellent electronic and spatial structures [33,34]. The p-type semiconductor Co_3_O_4_ exhibits excellent visible-light absorption properties. When it is integrated with an n-type semiconductor, a p–n type heterojunction can be constructed to upgrade the photocatalytic activity. Additionally, Co_3_O_4_ combines three desirable attributes—competitive pricing, environmental benignity, and suitable electronic properties—collectively qualifying it as an excellent heterojunction component. Wang et al. [35] synthesized a Co_3_O_4_/TiO_2_ p-n heterojunction photocatalyst, achieving a maximized hydrogen evolution of 3.46 mmol g^−1^ h^−1^, with significantly enhanced stability compared with pure TiO_2_ fibers. Ma et al. [36] synthesized a p–n heterojunction composite of Co_3_O_4_/CeO_2_, using a hydrothermal method, with an optimal H_2_ production rate of 2298.52 μmol g^−1^ h^−1^. Experimental results demonstrate that constructing a p–n heterojunction effectively enhances photocatalytic activity for the H_2_ evolution. When p-type Co_3_O_4_ with a comparatively narrow band gap of 1.2~2.1 eV [37,38,39,40] combines with the n-type semiconductor Zn_x_Cd_1−x_S known for its tunable electronic structure, the constructed p–n heterojunction delivers greatly improved photocatalytic activity under visible-light irradiation. So far, the Zn_x_Cd_1−x_S-based p–n heterojunction composite has been rarely reported despite its promising potential for highly efficient photocatalytic activity.

In this study, a series of Zn_x_Cd_1−x_S (0 ≤ x ≤ 1) was systematically investigated to probe the influence of compositional tuning on photocatalytic hydrogen evolution activity. The photocatalytic H_2_ evolution rates of Zn_x_Cd_1−x_S are shown in Figure S2. The experimental results demonstrate that the Zn_0.4_Cd_0.6_S solid solution achieves the optimal photocatalytic hydrogen evolution (8.36 mmol g^−1^ h^−1^). Based on this, the Zn_0.4_Cd_0.6_S solid solution was selected as the substrate catalyst. Parallel studies were conducted to evaluate the effects of gradient Co_3_O_4_ loading on the catalytic performance of the composite. Detailed characterizations of the composition, structure, morphology, BET surface area, and electrochemical and photoelectrochemical properties of the Co_3_O_4_/Zn_0.4_Cd_0.6_S composite were undertaken. Additionally, an in-depth exploration of the charge transfer process and the proposed photocatalytic mechanism was carried out. This study effectively identified the optimal band structure and Co_3_O_4_ loading amount for constructing the high-efficiency, noble-metal-free Co_3_O_4_/Zn_0.4_Cd_0.6_S p–n heterojunction composite, where the 4 wt%-Co_3_O_4_/Zn_0.4_Cd_0.6_S composite exhibits an optimal H_2_ evolution rate of 56.63 mmol g^−1^ h^−1^, approximately 6.8 times that of pure Zn_0.4_Cd_0.6_S and 2.9 times that of the 1.0 wt%-Pt/Zn_0.4_Cd_0.6_S composite (19.72 mmol g^−1^ h^−1^). The photocatalytic H_2_ evolution rate of the as-synthesized composite significantly outperforms that of many Co_3_O_4_/Zn_x_Cd_1−x_S heterojunctions and comparable photocatalysts reported in the literature, even exceeding that of certain noble-metal-modified composites (Table S4) [37,41,42,43,44,45,46,47,48,49,50,51,52,53,54,55,56,57,58]. Distinct from previous research on CZS/Co_3_O_4_ photocatalysts [59], this work systematically explores the influence of Zn/Cd molar ratios, quantifies actual Zn and Cd contents to establish a reliable composition–activity relationship, and adopts photodeposition experiments to directly verify the directional migration of photogenerated carriers across the heterojunction. In addition, systematic investigations into the photocatalytic mechanism reveal that the well-established p–n heterojunction with built-in electric field fundamentally accelerates photoinduced carriers’ separation and migration for high-efficiency H_2_ production. Meanwhile, the photocatalytic H_2_ evolution rate of the as-synthesized composite surpasses that of the reported photocatalyst.

## 2. Materials and Methods

### 2.1. Synthesis of Zn_x_Cd_1−x_S

Zn_x_Cd_1−x_S (0 ≤ x ≤ 1) was prepared via a solvothermal method following the protocol described in the relevant literature [60]. A mixture of 10 mL of H_2_O and 40 mL of ethylenediamine was used to dissolve 3(1−x) mmol of Cd(CH_3_COO)_2_·2H_2_O and 3x mmol of Zn(NO_3_)_2_·6H_2_O. Subsequently, 10 mL of an aqueous solution containing 0.4 M Na_2_S·9H_2_O was introduced, and the resulting mixture was stirred for 30 min. The synthesis was conducted hydrothermally (200 °C, 24 h) in sealed reactors. The products were isolated via centrifugation, washed with water and ethanol until pH neutrality was achieved, and had their moisture removed under vacuum desiccation (60 °C, 12 h). The molar ratio of Zn^2+^/Cd^2+^ in Zn_x_Cd_1−x_S (0 ≤ x ≤ 1) was quantified through ICP-OES analysis (Table S1).

### 2.2. Synthesis of Co_3_O_4_/Zn_0.4_Cd_0.6_S

An amount of 0.5 g of Zn_0.4_Cd_0.6_S was dispersed in 15 mL of DI H_2_O in a glass beaker and then homogenized with 30 mL of ethanol under vigorous ultrasonication for 0.5 h. Subsequently, varying amounts of Co(NO_3_)_2_·6H_2_O (0.0181, 0.0363, 0.0544, 0.0725, 0.0906, 0.1088, and 0.1269 g) were individually introduced into the above suspension under magnetic stirring. Aqueous ammonia was then titrated dropwise under continuous stirring, with a PHS-3E pH meter (INESA Scientific Instrument Co., Ltd., Shanghai, China) providing real-time readings to ensure the system was precisely adjusted to and stabilized at pH 9.0. The suspension was thereafter introduced into a 50 mL Teflon-lined autoclave, and the hydrothermal reaction ran continuously at 180 °C for 12 h, followed by centrifugation. The products were sequentially washed with DI H_2_O and ethanol until pH neutrality, prior to final dehydration in a vacuum oven (60 °C, 24 h). In the absence of Zn_0.4_Cd_0.6_S, Co_3_O_4_ was synthesized according to the same procedure.

Details of reagents and chemicals, characterization, photocatalytic activity test, and photoelectrochemical measurements of the as-prepared products are shown in the Supplementary Information (SI).

### 2.3. Photodeposition of Pt

An amount of 0.1 g of photocatalyst powder was loaded into a 100 mL reactor, and 50 mL of 5 vol% aqueous methanol solution was subsequently introduced. Argon gas was purged through the resultant suspension for 30 min to eliminate dissolved oxygen within the system. In the absence of light, 1.71 mL of 3 mmol L^−1^ K_2_PtCl_4_ solution was pipetted into the mixture. The resulting slurry was magnetically stirred for another 30 min under dark conditions. The mixture was maintained at ambient temperature using a water bath and exposed to visible light from a 300 W Xe lamp (420 nm) (Beijing MerryChange Technology Co., Ltd., Beijing, China) for 30 min with constant stirring. Once the illumination terminated, the solid precipitate was harvested by centrifugation. After collection, the solid precipitates underwent repeated rinsing using deionized water and ethanol and were then dried under vacuum at 60 °C.

## 3. Results and Discussion

### 3.1. Composition and Crystalline Phase Analysis

**Figure 1:**
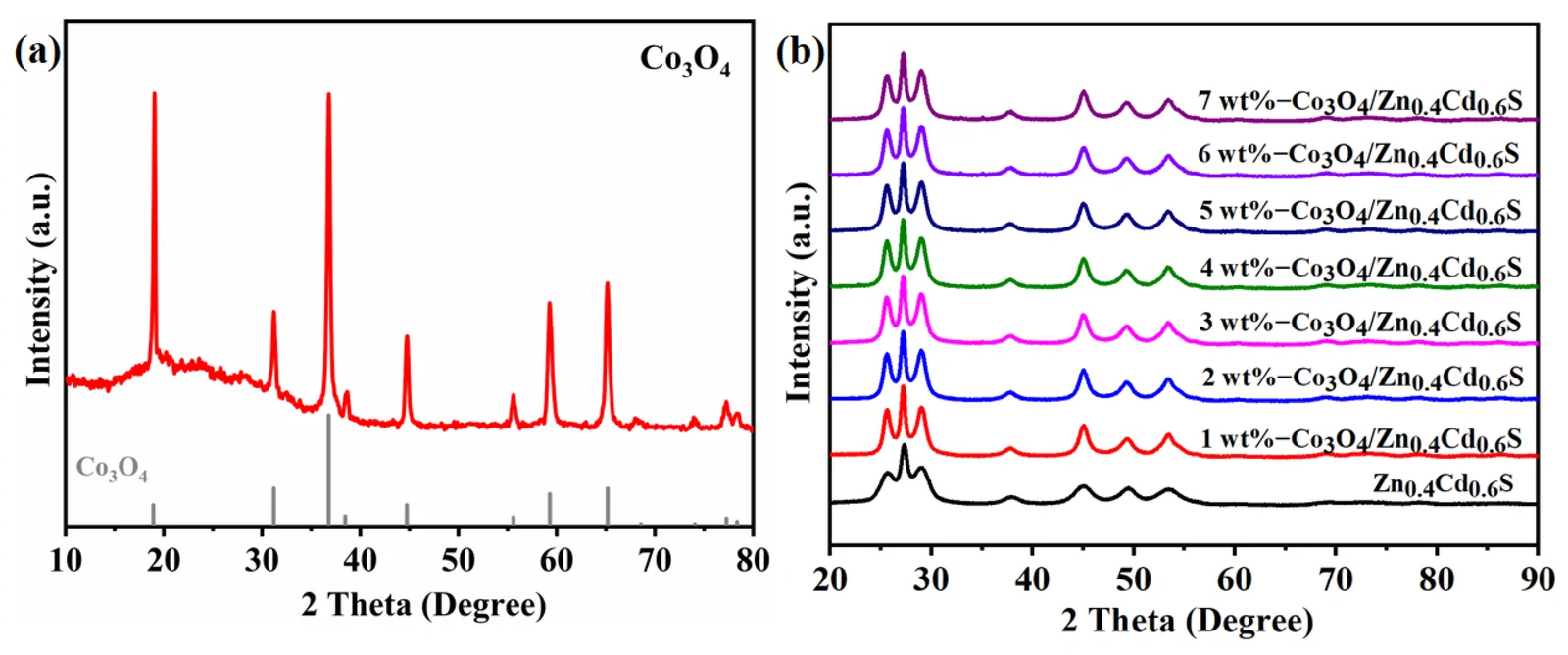
XRD patterns: (**a**) cubic Co_3_O_4_ and (**b**) Co_3_O_4_/Zn_0.4_Cd_0.6_S composites.

Figure 1 illustrates XRD patterns of Co_3_O_4_ and Co_3_O_4_/Zn_0.4_Cd_0.6_S. Figure 1a shows that several distinct diffraction peaks correspond to crystal faces of cubic Co_3_O_4_ (JCPDS 42-1467), respectively [61,62,63]. It is confirmed that the as-synthesized product is pure Co_3_O_4_. X-ray diffractograms of Zn_x_Cd_1−x_S (0 < x < 1) exhibit full agreement with the standard hexagonal Cd_0.8_Zn_0.2_S solid solution reference patterns (JCPDS 49-1302-10), confirming solid solution formation (Figure S1) [60,64]. The XRD patterns of Co_3_O_4_/Zn_0.4_Cd_0.6_S with different mass ratios of Co_3_O_4_ (Figure 1b) clearly show the characteristic diffraction peaks of Zn_0.4_Cd_0.6_S in the composite, which are slightly offset compared with those of pure Zn_0.4_Cd_0.6_S; this may be due to the interaction between Co_3_O_4_ and Zn_0.4_Cd_0.6_S. The results indicate that the crystal structure of Zn_0.4_Cd_0.6_S remains unaltered upon the incorporation of Co_3_O_4_.

Table 1 summarizes the mass percentage of Co_3_O_4_ in the Co_3_O_4_/Zn_0.4_Cd_0.6_S composites, as determined via ICP-OES analysis. It is shown that the actual loading content (mass percentage) of Co_3_O_4_ is closely aligned with the feed ratio, and thus, the mass percentages of Co_3_O_4_ in the composite are denoted as 1, 2, 3, 4, 5, 6, and 7 wt% for clarity.

### 3.2. XPS Analysis

**Figure 2:**
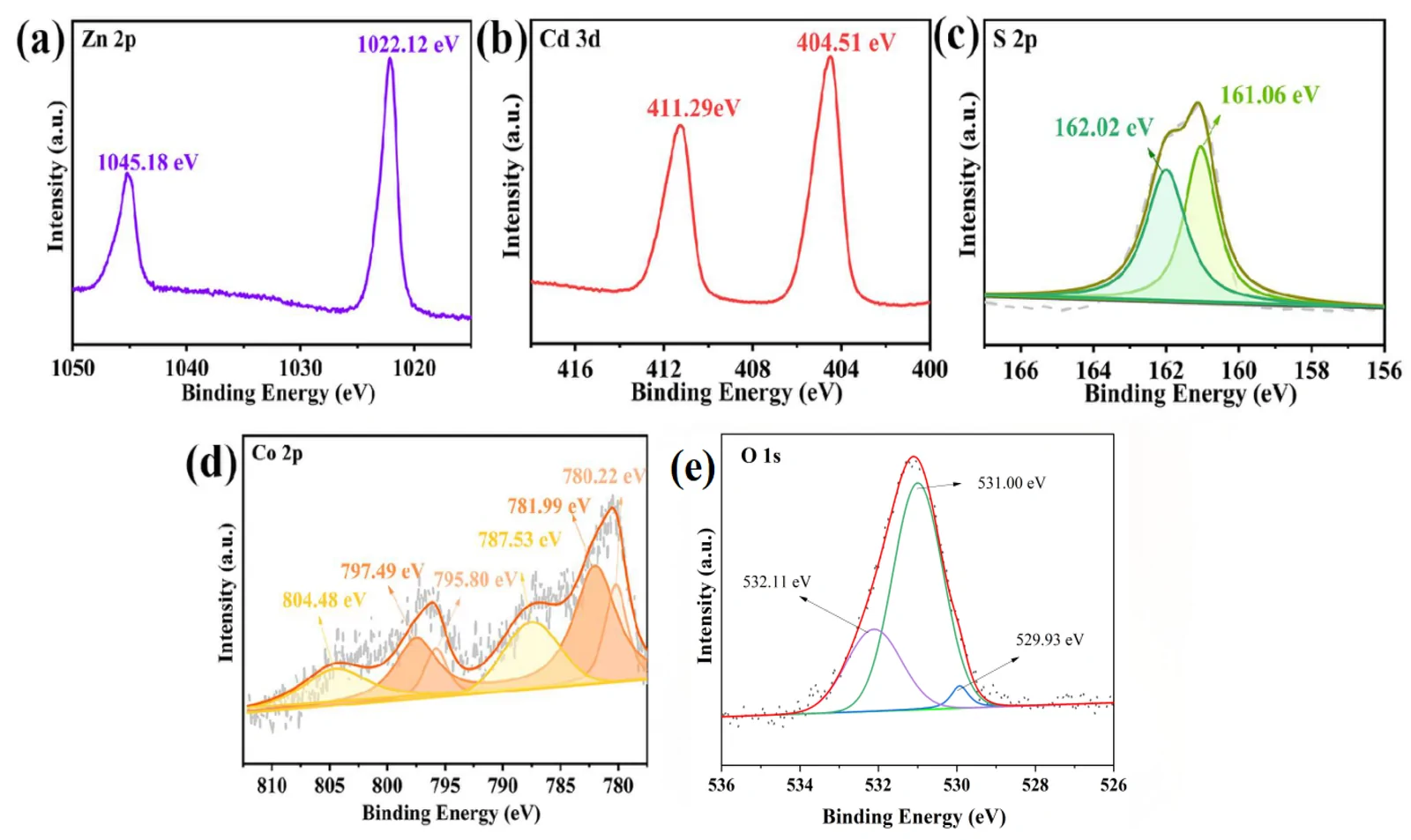
XPS of Co_3_O_4_/Zn_0.4_Cd_0.6_S: (**a**) Zn 2p, (**b**) Cd 3d, (**c**) S 2p, (**d**) Co 2p, (**e**) O 1s.

Figure 2 displays the XPS spectra of Co_3_O_4_/Zn_0.4_Cd_0.6_S, which clearly demonstrate the coexistence of Co, O, Zn, Cd, and S elements. Figure 2a illustrates that the Zn 2p XPS spectrum exhibits characteristic peaks at binding energies of 1022.12 and 1045.18 eV, corresponding to Zn 2p_3/2_ and Zn 2p_1/2_, respectively, indicating the presence of Zn^2+^ species in the composite. The Cd 3d spectrum (Figure 2b) exhibits binding energies of 404.51 eV (Cd 3d_5/2_) and 411.29 eV (Cd 3d_3/2_), indicating the presence of Cd^2+^. The S 2p XPS spectrum (Figure 2c) shows two well-resolved peaks centered at 161.06 and 162.02 eV, which can be assigned to S 2p_3/2_ and S 2p_1/2_, respectively, demonstrating the presence of S^2−^ in the sulfide lattice. The measured binding energies for Zn, Cd, and S exhibit close alignment with established reference values [65]. Four binding energy values are extracted from the Co 2p XPS spectrum (Figure 2d)—780.22 eV and 795.80 eV for Co^3+^ 2p_3/2_ and Co^3+^ 2p_1/2_, contrasted with 781.99 and 797.49 eV for Co^2+^ counterparts—confirming the coexistence of Co^3+^ and Co^2+^ species. Additionally, two distinct satellite peaks are detected at 787.53 eV and 804.48 eV, in line with previously reported values for Co_3_O_4_, further supporting the presence of this phase in the composite [66,67]. In addition, the peak at 529.93 eV is assigned to lattice oxygen (Co–O) in the Co_3_O_4_ spinel structure; the dominant component at 531.00 eV is attributed to surface hydroxyl species and low-coordinated surface oxygen, and the component at 532.11 eV corresponds to surface-adsorbed water [68,69,70]. The lattice oxygen component accounts for only ~3% of the total O 1s area, which is consistent with the low Co_3_O_4_ loading (4 wt%) in the composite and the surface sensitivity of XPS. All the above results further confirm that the composite comprises Co_3_O_4_ and Zn_0.4_Cd_0.6_S.

### 3.3. Morphology Analysis

**Figure 3:**
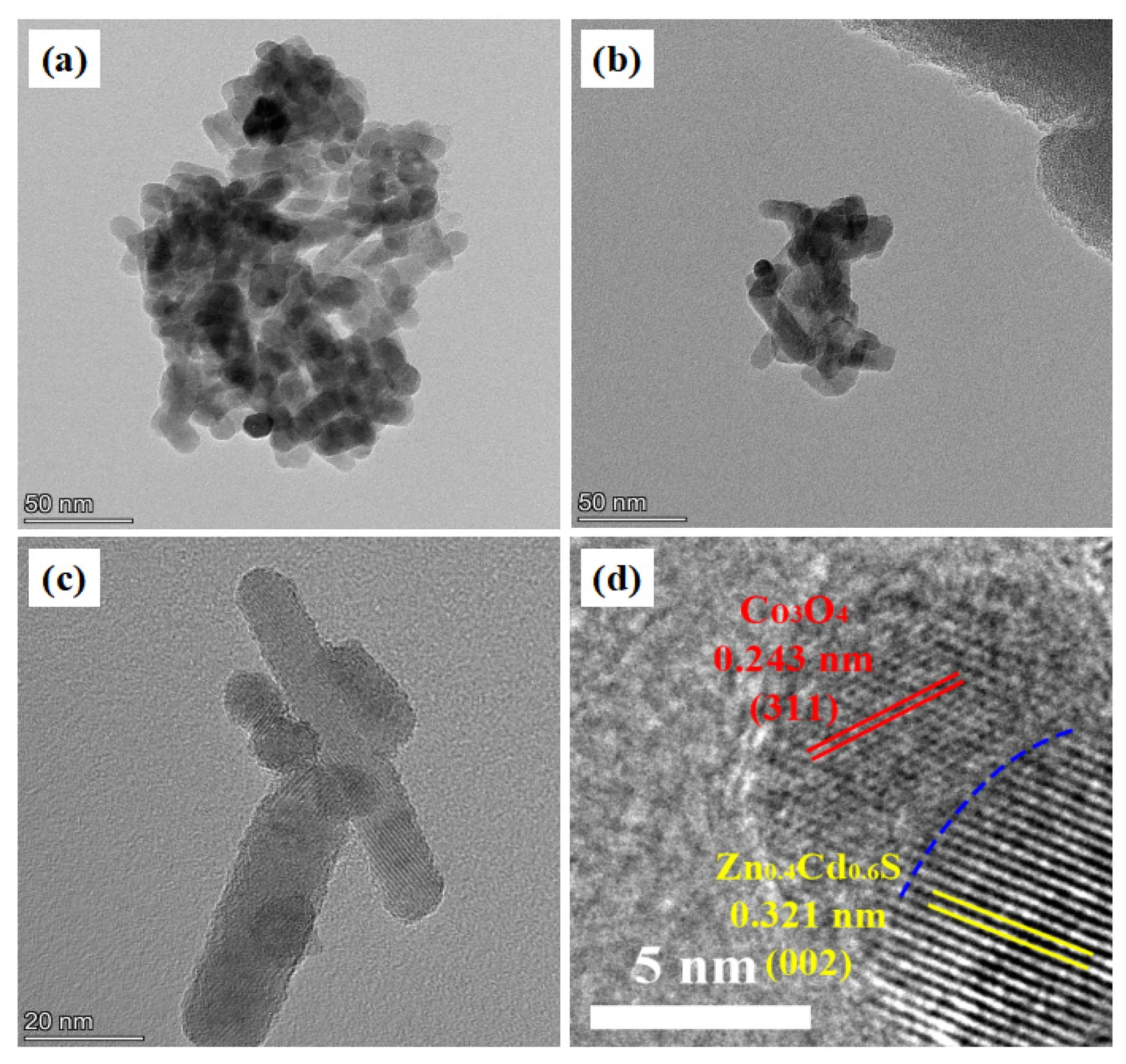
TEM (**a**,**b**) and HRTEM (**c**,**d**) images of Co_3_O_4_/Zn_0.4_Cd_0.6_S composite. The blue dashed line in (**d**) marks the heterointerface between Co_3_O_4_ and Zn_0.4_Cd_0.6_S.

Figure 3 presents the TEM (Figure 3a,b) and HRTEM (Figure 3c,d) images of Co_3_O_4_/Zn_0.4_Cd_0.6_S. The surface of Zn_0.4_Cd_0.6_S nanorods is closely adhered with Co_3_O_4_ nanoparticles. It reveals the contact interfaces between Co_3_O_4_ and Zn_0.4_Cd_0.6_S, which facilitates photoexcited charge separation and migration [71]. Figure 3d shows that the interplanar spacings of 0.243 and 0.321 nm are assigned to the (311) plane of cubic Co_3_O_4_ and (002) plane of hexagonal Zn_0.4_Cd_0.6_S, respectively. The intimate contact between the lattice fringes of Co_3_O_4_ and Zn_0.4_Cd_0.6_S demonstrates the formation of close heterogeneous interfaces, which efficiently promote photogenerated charge carriers separation and transfer [33,34].

**Figure 4:**
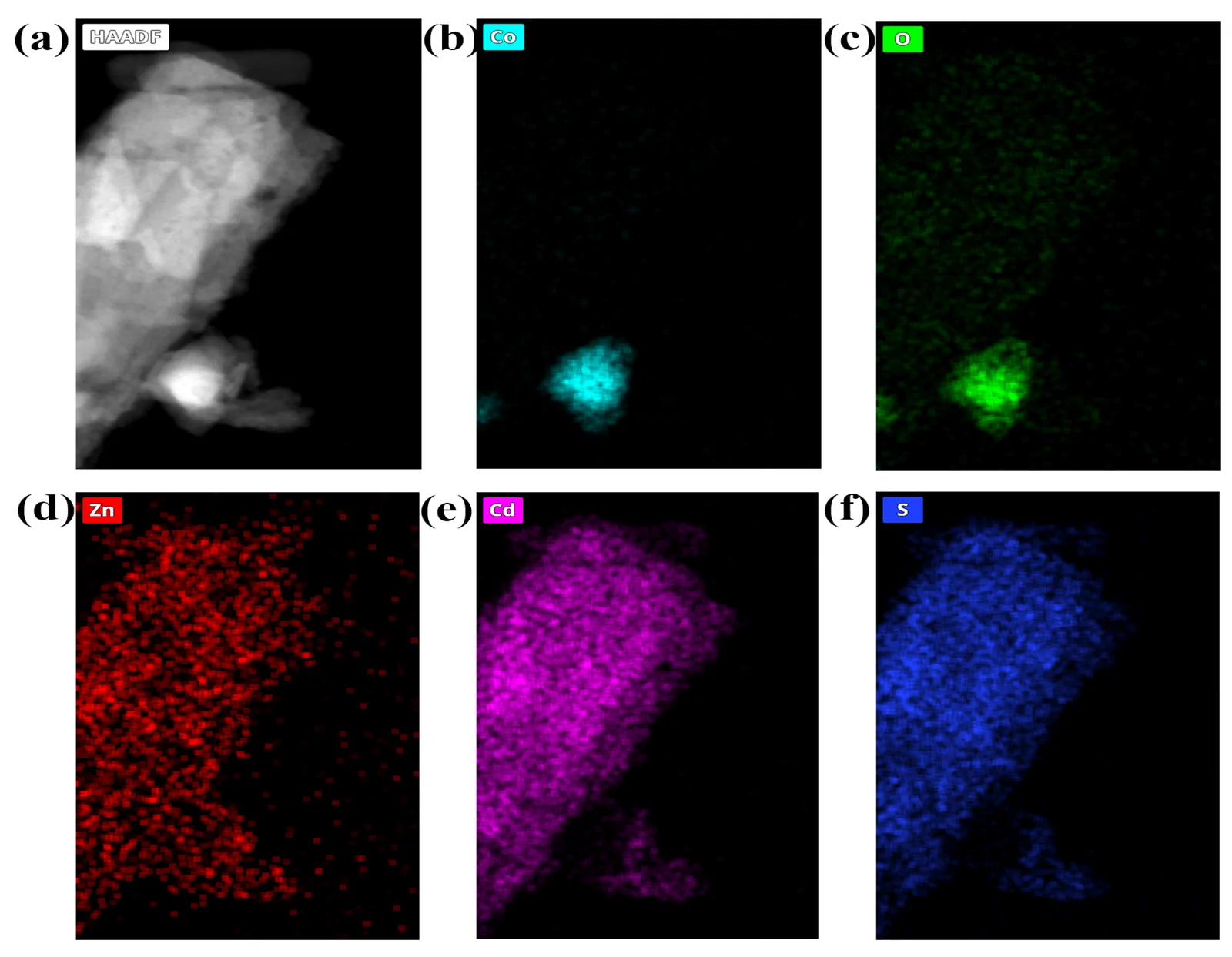
HAADF-STEM image of Co_3_O_4_/Zn_0.4_Cd_0.6_S composite (**a**) and elemental mapping images of Co (**b**), O (**c**), Zn (**d**), Cd (**e**), and S (**f**).

Figure 4 exhibits the HAADF-STEM elemental mapping images of the as-prepared composite, suggesting that the distribution of Zn, Cd, S, Co, and O elements in the structure of Co_3_O_4_/Zn_0.4_Cd_0.6_S, and Co_3_O_4_ nanoparticles is dispersed on the surface of Zn_0.4_Cd_0.6_S nanorods. This result verifies the formation of the heterojunction between Co_3_O_4_ and Zn_0.4_Cd_0.6_S. By integrating the analytical results of XRD, XPS, TEM, and HRTEM, the formation of Co_3_O_4_ is further confirmed, with its dispersion on the Zn_0.4_Cd_0.6_S surface enabling the construction of the heterojunction between the two components.

**Figure 5:**
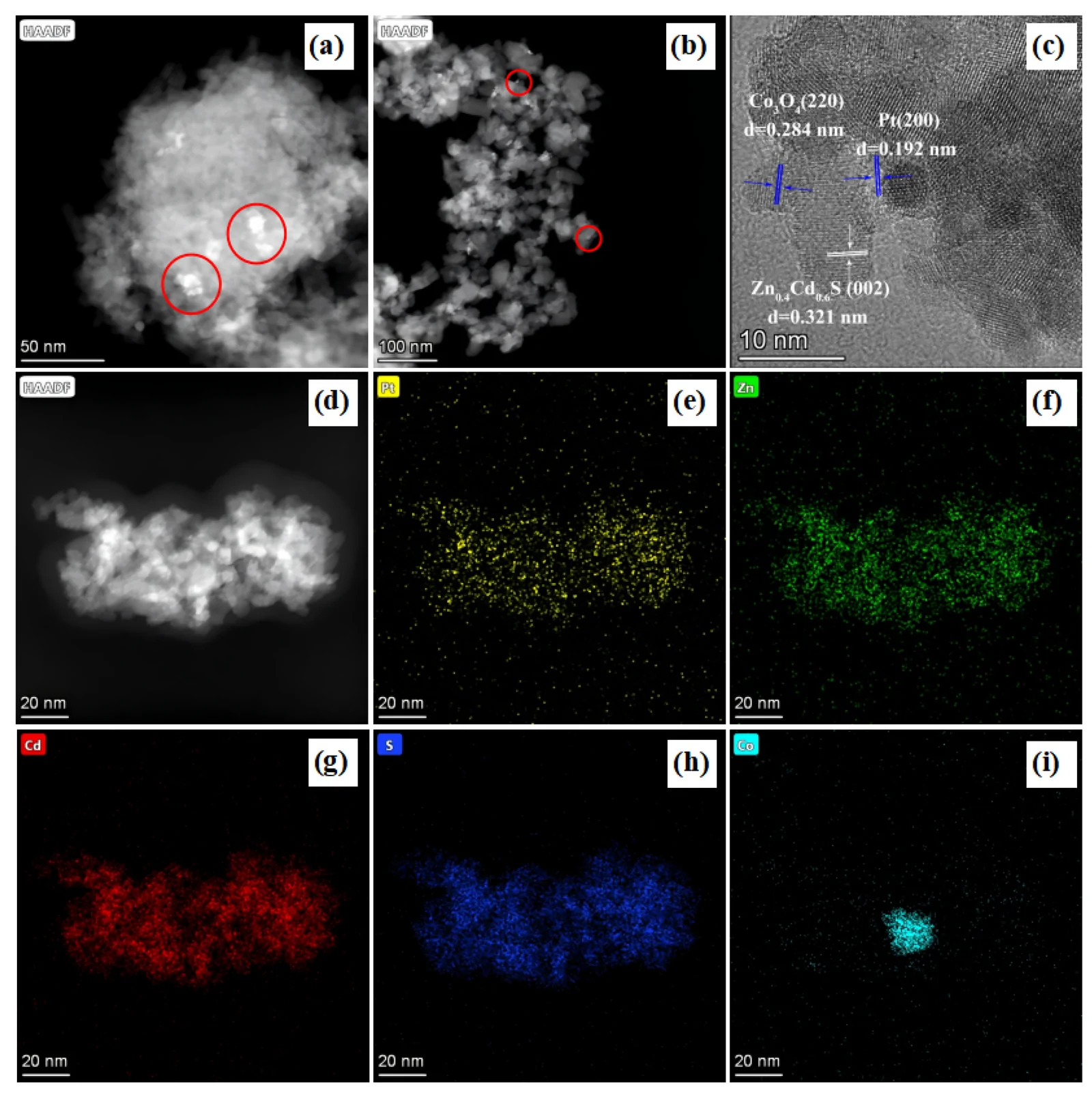
HAADF-STEM images of Pt-photodeposited Zn_0.4_Cd_0.6_S (**a**) and Co_3_O_4_/Zn_0.4_Cd_0.6_S composite (**b**,**d**); HRTEM image (**c**) and corresponding elemental mapping of Pt (**e**), Zn (**f**), Cd (**g**), S (**h**), and Co (**i**) for the Pt-photodeposited composite. The red circles in panels (**a**,**b**) mark the photodeposited Pt nanoparticles.

To directly visualize the directional charge migration induced by the coupling of Co_3_O_4_ and Zn_0.4_Cd_0.6_S, photodeposition experiments were performed. TEM, HRTEM and HAADF-STEM images were employed to investigate the location and distribution of photodeposited Pt nanoparticles on the sample surface. Figure 5a,b presents HAADF-STEM images of Pt-photodeposited Zn_0.4_Cd_0.6_S (a) and the Co_3_O_4_/Zn_0.4_Cd_0.6_S composite (b), showing the locations and distributions of Pt nanoparticles after 30 min of photocatalytic H_2_ evolution. As can be seen from Figure 5b, Pt nanoparticles are mostly situated on the Zn_0.4_Cd_0.6_S portion of the composite, and this observation is validated via HAADF-STEM elemental mapping (Figure 5e–i). Compared with Zn_0.4_Cd_0.6_S (Figure 5a), photodeposited Pt nanoparticles on the composite display smaller sizes and more uniform spatial distribution (Figure 5b). Figure 5c shows that the interplanar spacings of 0.192, 0.284 and 0.321 nm are assigned to the (200) plane of cubic Pt, the (220) plane of cubic Co_3_O_4_ and the (002) plane of hexagonal Zn_0.4_Cd_0.6_S, respectively. It can be seen from Figure 5c that Pt nanoparticles are preferentially deposited on the surface of Zn_0.4_Cd_0.6_S, and an intimate contact interface exists between the lattice fringes of Pt and Zn_0.4_Cd_0.6_S. These experimental findings confirm that the constructed heterojunction boosts the oriented transport of light-generated charge carriers [72]. Upon visible-light illumination, photogenerated electrons migrate to Zn_0.4_Cd_0.6_S in the composite, converting Pt precursor species into metallic Pt and resulting in site-specific Pt deposition.

### 3.4. BET Surface Area and Aperture Distribution

**Figure 6:**
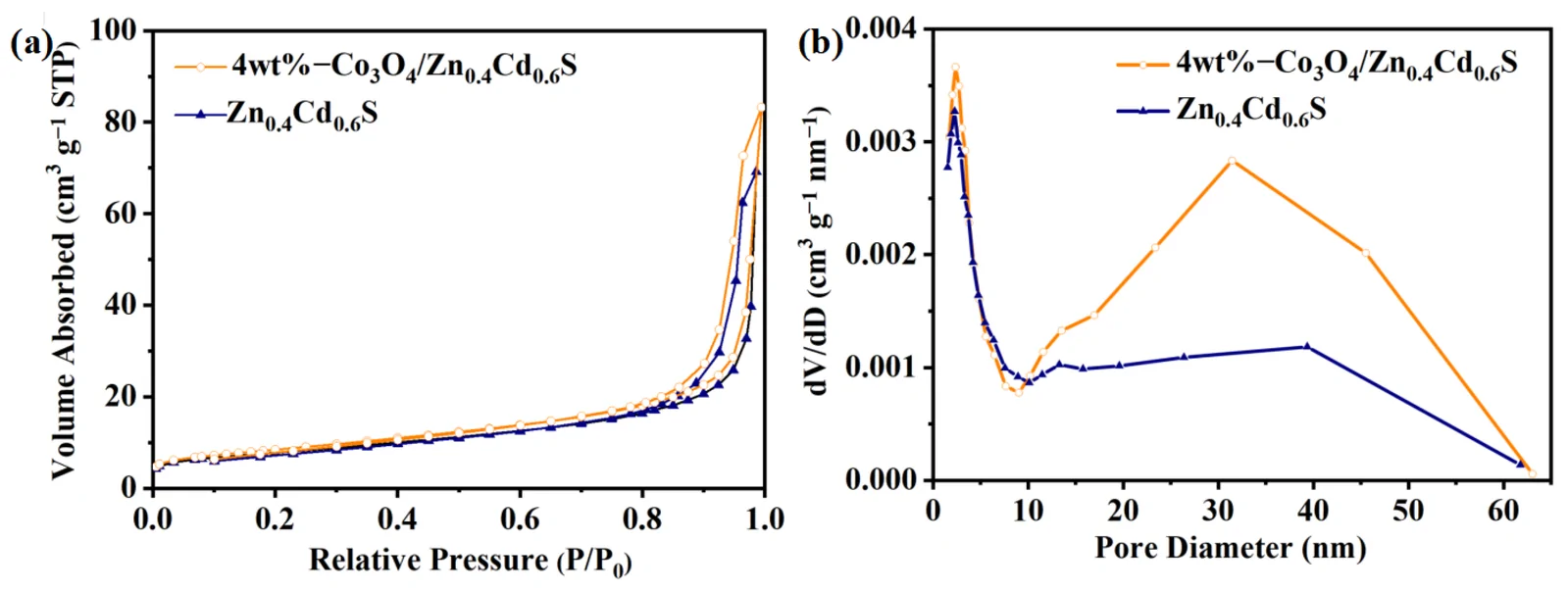
(**a**) Nitrogen adsorption–desorption isotherms and (**b**) pore size distributions of Zn_0.4_Cd_0.6_S and Co_3_O_4_/Zn_0.4_Cd_0.6_S.

The comparative N_2_ adsorption–desorption and pore size profiles for Zn_0.4_Cd_0.6_S versus Co_3_O_4_/Zn_0.4_Cd_0.6_S are displayed in Figure 6. In accordance with the IUPAC classification, the two measured samples show the classic type-IV isotherm profiles featuring H_3_ hysteresis loops. (P/P0: 0.9–1.0) (Figure 6a), indicating the coexistence of mesoporosity and macroporosity in the samples [73,74,75,76]. The pore size distributions of Zn_0.4_Cd_0.6_S and Co_3_O_4_/Zn_0.4_Cd_0.6_S are exhibited in Figure 6b, with ranges of 2–62 nm for Zn_0.4_Cd_0.6_S and 2–63 nm for Co_3_O_4_/Zn_0.4_Cd_0.6_S, which further demonstrates the presence of mesopores and macropores in the two samples. An apparent BET surface area increase from 25.61 m^2^ g^−1^ (Zn_0.4_Cd_0.6_S) to 30.39 m^2^ g^−1^ was observed for the Co_3_O_4_/Zn_0.4_Cd_0.6_S composite; this may be due to the homogeneous loading of Co_3_O_4_ nanoparticles onto Zn_0.4_Cd_0.6_S nanorods, resulting in an enlarged surface area, which enables access to more active sites for photocatalytic hydrogen generation, potentially leading to enhanced activity through improved reactant accessibility and surface-mediated redox processes.

### 3.5. UV–Vis DRS Analysis

**Figure 7:**
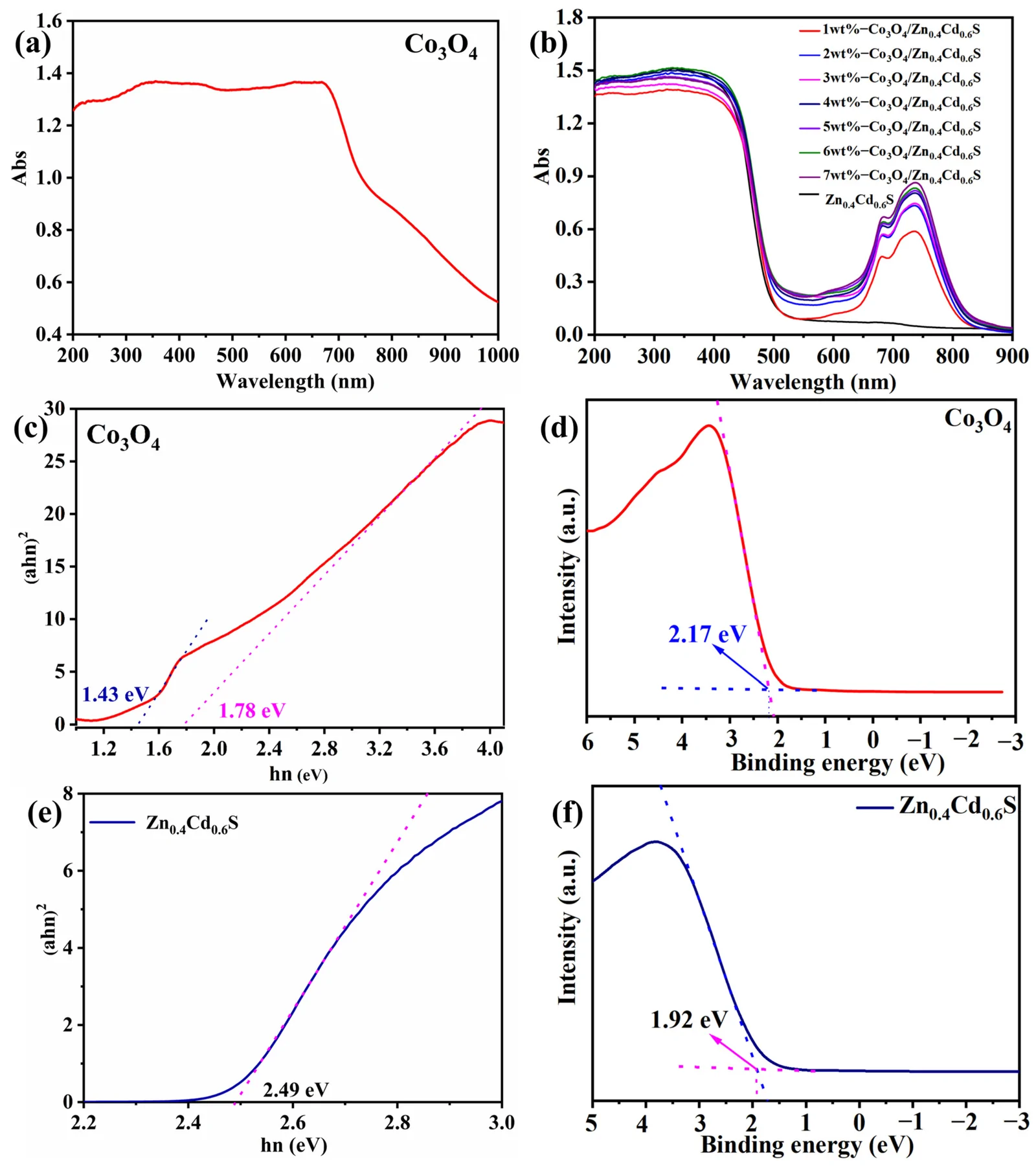
UV–Vis spectra of (**a**) Co_3_O_4_ and (**b**) Co_3_O_4_/Zn_0.4_Cd_0.6_S, (**c**) band gap of Co_3_O_4_, (**d**) VB-XPS spectrum of Co_3_O_4_, (**e**) band gap of Zn_0.4_Cd_0.6_S, and (**f**) VB-XPS spectrum of Zn_0.4_Cd_0.6_S.

UV–Vis diffuse reflectance spectroscopy was employed to quantify the light absorption properties of Co_3_O_4_, Zn_0.4_Cd_0.6_S, and Co_3_O_4_/Zn_0.4_Cd_0.6_S (Figure 7). Co_3_O_4_, with a narrow band gap, exhibits broad absorption peaks in the visible-light and near-infrared regions (Figure 7a). Zn_0.4_Cd_0.6_S exhibits an absorption edge at about 501 nm, and the absorption edge of Co_3_O_4_/Zn_0.4_Cd_0.6_S gradually shifts to 510 nm with the increase in Co_3_O_4_ content (Figure 7b); this may be due to the close contact at Co_3_O_4_ and Zn_0.4_Cd_0.6_S interfaces [37]. Additionally, two progressively enhanced acromial peaks located within 550–850 nm are observed in the composites with increasing Co_3_O_4_ content, which are assigned to optical transition signatures of Co_3_O_4_ [77,78], indicating the presence of Co_3_O_4_. The results demonstrate that incorporating Co_3_O_4_ significantly enhances the visible-light absorption capacity of the composite, leading to increased generation of photogenerated electrons that effectively promote photocatalytic hydrogen evolution.

As estimated by the Kubelka–Munk formula [79], the band gap values of Co_3_O_4_ are *E*g_1_ = 1.43 eV and *E*g_2_ = 1.78 eV (Figure 7c), which are assigned to the charge transitions of O^2−^→Co^3+^ and O^2−^→Co^2+^, respectively [77,79,80,81,82]. The electronic structure of Co_3_O_4_ is primarily governed by Co^2+^ 3d and O^2−^ 2p orbitals, which correspond to the conduction band (CB) and valence band (VB), respectively, while the Co^3+^ energy band serves as a secondary band. Analytical data confirm a 1.78 eV band gap for Co_3_O_4_. Figure 7d shows that the VB-XPS spectrum yields a valence band potential (*E*_VB_) of 2.17 eV for Co_3_O_4_, with the corresponding conduction band potential (*E*_CB_) calculated to be 0.39 eV when using the relation *E*_CB_ = *E*_VB_ − *E*_g_ [83]. For Zn_0.4_Cd_0.6_S, the band gap is 2.49 eV (Figure 7e), with an *E*_VB_ of 1.92 eV (Figure 7f). Applying the same method, the *E_CB_* is determined to be −0.57 eV. The narrower bandgap of Co_3_O_4_ compared with Zn_0.4_Cd_0.6_S extends the visible-light absorption range of the composite. The augmented photon absorption capability significantly increases the yield of electron–hole pairs. Concurrently, the refined charge carrier kinetics (including efficient separation and rapid transportation) synergistically lead to remarkable improvement in hydrogen production efficiency. These band gap measurements provide crucial experimental evidence for exploring the charge transfer mechanism.

### 3.6. Photocatalytic H_2_ Production

**Figure 8:**
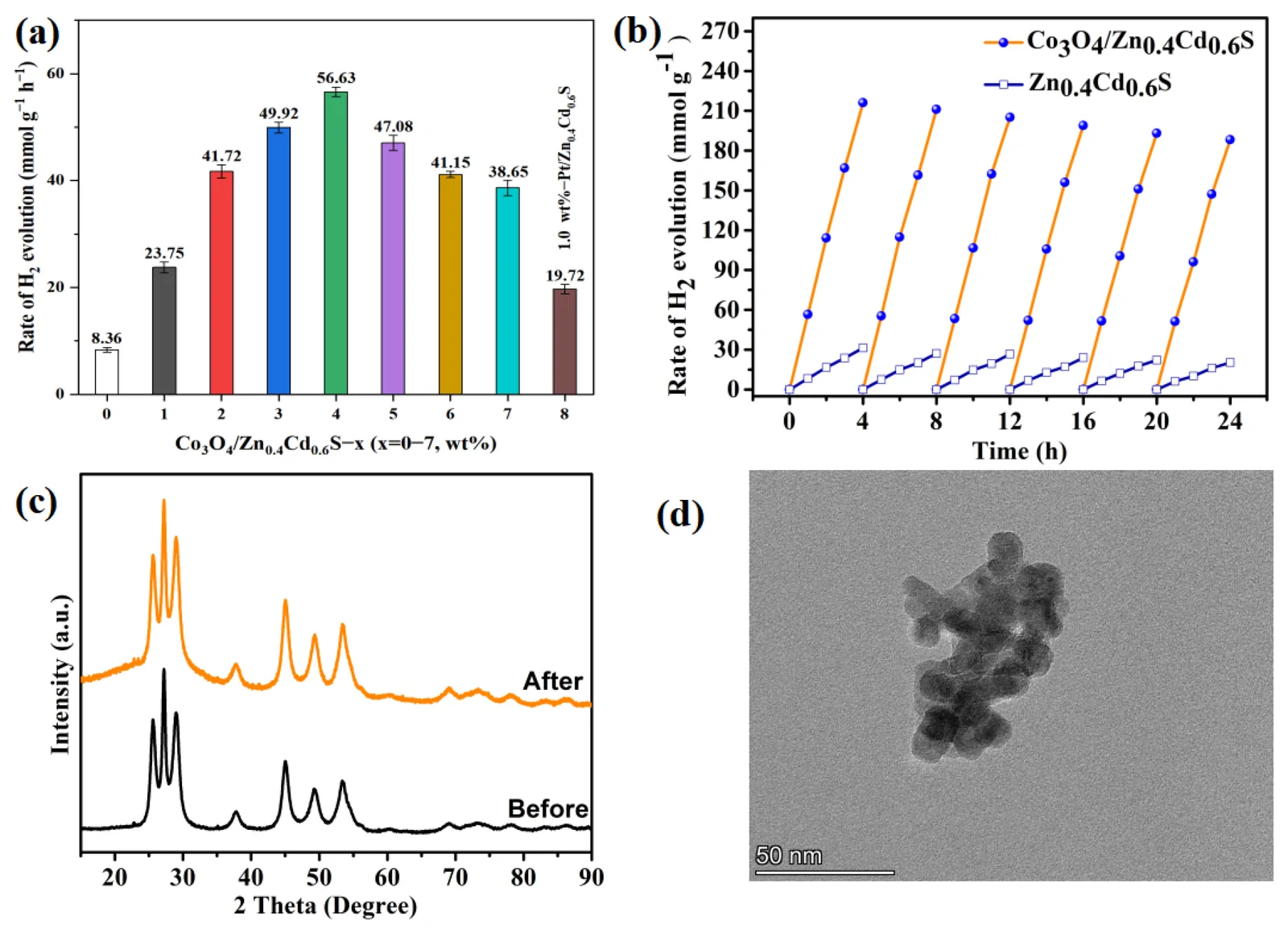
(**a**) Comparison of the photocatalytic H_2_ evolution rate of Co_3_O_4_/Zn_0.4_Cd_0.6_S-x (x = 0–7, wt%) and 1.0 wt%-Pt/Zn_0.4_Cd_0.6_S. (**b**) Cyclic stability test of photocatalytic H_2_ evolution on Zn_0.4_Cd_0.6_S and Co_3_O_4_/Zn_0.4_Cd_0.6_S, (**c**) XRD of Co_3_O_4_/Zn_0.4_Cd_0.6_S before and after the reaction, and (**d**) TEM of Co_3_O_4_/Zn_0.4_Cd_0.6_S after the recycling experiments. All presented results were obtained by averaging three independent trials. Standard deviations derived from the three parallel measurements are denoted by the error bars.

The photocatalytic H_2_ production rate of Co_3_O_4_/Zn_0.4_Cd_0.6_S with varying mass ratios of Co_3_O_4_ was evaluated in a 0.5 M Na_2_S/Na_2_SO_3_ sacrificial agent solution exposed to visible spectrum illumination (λ ≥ 420 nm). When Co_3_O_4_ was used alone as the photocatalyst, H_2_ was not detected in the experiment. After loading in situ Co_3_O_4_ onto Zn_0.4_Cd_0.6_S to form the Co_3_O_4_/Zn_0.4_Cd_0.6_S composite, the H_2_ evolution rate of the Co_3_O_4_/Zn_0.4_Cd_0.6_S composite increases significantly (Figure 8a). The results demonstrate that incorporating Co_3_O_4_ significantly enhances the photocatalytic activity of the composite. With merely 1 wt% Co_3_O_4_ loading, the hydrogen generation capacity of the composite reaches 23.75 mmol g^−1^ h^−1^, outperforming the unmodified Zn_0.4_Cd_0.6_S (8.36 mmol g^−1^ h^−1^) by a factor of 2.8. Notably, when the Co_3_O_4_ content is increased to 4 wt%, Co_3_O_4_/Zn_0.4_Cd_0._6S demonstrates exceptional hydrogen production performance (56.63 mmol g^−1^ h^−1^), exhibiting approximately 6.8 times higher activity than pure Zn_0.4_Cd_0.6_S and 2.9 times that of the 1.0 wt%-Pt/Zn_0.4_Cd_0.6_S composite (19.72 mmol g^−1^ h^−1^). This performance surpasses that of many comparable photocatalysts reported in the literature and even exceeds that of certain noble-metal-modified composites (Table S4) [37,41,42,43,44,45,46,47,48,49,50,51,52,53,54,55,56,57,58]. The measured photocatalytic hydrogen generation rates correspond to averages derived from three independent parallel trials. Data regarding standard deviation (SD) and relative standard deviation (RSD) are compiled in Table S2 of the Supplementary Information. The results reveal that the RSD values of photocatalytic H_2_ evolution rates for the as-synthesized products are 1.53%, confirming good measurement reproducibility. The statistical summary of photocatalytic H_2_ evolution rates and Welch’s *t*-test results is provided in Table S3 of the Supplementary Information, suggesting that the difference in photocatalytic H_2_ evolution performance between Zn_0.4_Cd_0.6_S and the composite is statistically significant (*p* < 0.05). The AQE of the as-synthesized composite is 21.86% at 420 nm. When the loading is within 4 wt%, the photocatalytic activity of the composite rises with increasing loading. This can be ascribed to the formation of a Co_3_O_4_/Zn_0.4_Cd_0.6_S heterojunction, which effectively accelerates the separation and interfacial migration of photogenerated charge carriers. In addition, the progressively increased loading offers more active sites, further enhancing the photocatalytic hydrogen evolution performance. However, when the Co_3_O_4_ content of the composite increases past 4 wt%, the H_2_ evolution rate continually decreases, which may be due to the negative role of excessive Co_3_O_4_ as follows: on the one hand, beyond optimal loading thresholds, Co_3_O_4_ overlayers induce photon shielding effects that proportionally degrade the visible-light harvesting capability of Co_3_O_4_/Zn_0.4_Cd_0.6_S. This can be confirmed by the gradual color change of the composite from light yellow to dark green as the Co_3_O_4_ content increases. On the other hand, excessive Co_3_O_4_ may become recombination centers of photogenerated charge carriers [37]. Furthermore, excessive Co_3_O_4_ can cover the active sites of Zn_0.4_Cd_0.6_S, which inhibits photocatalytic hydrogen production. Consequently, 4 wt% Co_3_O_4_ loading is identified as the optimal composition for the Co_3_O_4_/Zn_0.4_Cd_0.6_S composite.

Photostability is a critical parameter for assessing photocatalytic performance. The cyclic stability of Co_3_O_4_/Zn_0.4_Cd_0.6_S was evaluated every 4 h as a cycle (Figure 8b). The total H_2_ production on Co_3_O_4_/Zn_0.4_Cd_0.6_S in the first cycle is 216.11 mmol g^−1^. After six cycles, the total H_2_ production is 188.23 mmol g^−1^, representing 87.1% of the total H_2_ production in the first cycle. In comparison, the total H_2_ production on pure Zn_0.4_Cd_0.6_S after six cycles is only 64.9% of that in the first cycle. It can be concluded that the introduction of Co_3_O_4_ dramatically enhances the photocatalytic activity and durability of the composite. ICP-OES measurements were performed on the composite after the recycling tests. The results show that the actual loading content (mass fraction) of Co_3_O_4_ after the tests is 3.89 wt%. Compared with the initial value (4.03 wt%), only a slight decrease in the loading amount is observed. Figure 8c shows the XRD patterns of Co_3_O_4_/Zn_0.4_Cd_0.6_S composite before and after the tests, and Figure 8d exhibits the TEM images of the composite after the recycling experiments. Dual-modal characterization integrating XRD (Figure 8c) and TEM (Figure 3b and Figure 8d) confirms no significant changes in crystal phase composition and nanoscale morphology in the composite after the recycling experiments compared with the pristine sample. TEM characterization of Zn_0.4_Cd_0.6_S before and after the recycling experiments is provided in Figure S3 of the Supplementary Information. In contrast, bare Zn_0.4_Cd_0.6_S exhibits substantial morphological changes according to the TEM images after the recycling tests. These experimental results further verify that the introduction of Co_3_O_4_ can efficiently suppress photocorrosion of the composite.

### 3.7. Separation of Photogenerated Charge Carriers

**Figure 9:**
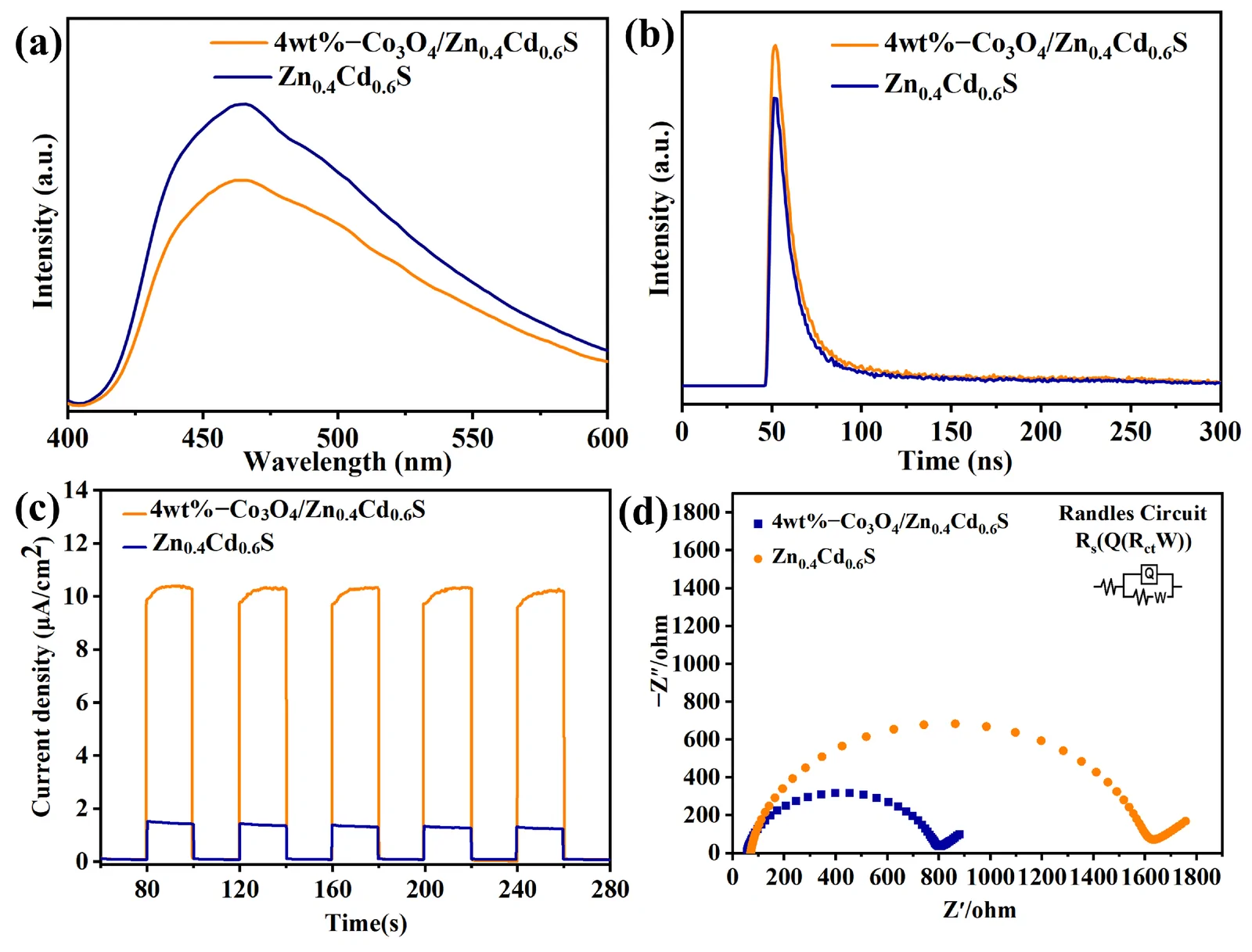
(**a**) PL spectra, (**b**) TRPL spectra, (**c**) transient photocurrent curves, and (**d**) EIS Nyquist plots of Zn_0.4_Cd_0.6_S and Co_3_O_4_/Zn_0.4_Cd_0.6_S.

PL spectra of Zn_0.4_Cd_0.6_S and Co_3_O_4_/Zn_0.4_Cd_0.6_S are shown in Figure 9a, collected using an excitation wavelength of 337 nm. Both samples exhibit prominent emission peaks around 463 nm, but the PL intensity of pure Zn_0.4_Cd_0.6_S is remarkably greater than that of Co_3_O_4_/Zn_0.4_Cd_0.6_S. In general, more intense PL emission peaks are indicative of a higher frequency of electron–hole pair recombination in the material, which reduces the number of active charge carriers available to participate in surface redox reactions, leading to diminished photocatalytic activity [42,73,84,85,86,87,88]. Characterization results demonstrate that the incorporated Co_3_O_4_ nanoparticles substantially inhibit charge recombination through the established heterojunction structure, leading to a marked improvement in photocatalytic performance.

TRPL spectra of pure Zn_0.4_Cd_0.6_S and the Co_3_O_4_/Zn_0.4_Cd_0.6_S composite are shown in Figure 9b. According to Formula (1), the average lifetimes of the charge carriers for Zn_0.4_Cd_0.6_S and Co_3_O_4_/Zn_0.4_Cd_0.6_S were calculated to be 9.66 and 10.44 ns, respectively [89,90]:

(1) $$\tau =\frac{\sum A_{n}{\tau_{n}}^{2}}{A_{n}\tau_{n}}\;(n=1,2,3\ldots \ldots )$$

The results reveal that the charge carrier lifetime in Co_3_O_4_/Zn_0.4_Cd_0.6_S is significantly extended compared with pristine Zn_0.4_Cd_0.6_S, confirming that modification with Co_3_O_4_ can restrain the recombination of photoinduced electron–hole pairs, which promotes the spatial dissociation and transport of photogenerated charges in the composite, thereby enhancing its catalytic performance in hydrogen production [42,73,84,85,86,87,91]. These experimental findings establish a direct structure–activity relationship, where the construction of heterojunctions effectively promotes electron–hole separation and migration, thereby greatly boosting the hydrogen production efficiency.

The photoelectrochemical properties of Zn_0.4_Cd_0.6_S and Co_3_O_4_/Zn_0.4_Cd_0.6_S samples were measured to further evaluate the interfacial charge transfer rate of the samples. Figure 9c presents the transient photocurrent responses of the investigated samples. Co_3_O_4_/Zn_0.4_Cd_0.6_S demonstrates a substantially higher photocurrent density compared with pristine Zn_0.4_Cd_0.6_S after simulated visible-light (λ ≥ 420 nm) irradiation. Notably, the composite maintains excellent photocurrent stability, with negligible degradation observed over multiple on–off cycles. This enhancement is primarily attributed to the well-constructed p–n heterojunction at the Co_3_O_4_/Zn_0.4_Cd_0.6_S interface, which enables efficient charge transport and recombination inhibition, resulting in considerable improvements in photoelectrochemical behavior [37,42,77].

The EIS spectra of both Zn_0.4_Cd_0.6_S and the Co_3_O_4_/Zn_0.4_Cd_0.6_S composite have been fitted using the equivalent circuit *R*_s_(*Q*(*R*_ct_*W*)) (Figure 9d) with ZSimpWin software (Version 3.60, EChem Software, Warminster, PA, USA). *R*_s_ refers to the resistance of the electrolyte solution, and *R*_ct_ denotes the charge transfer resistance, while *Q* is assigned as a constant phase element (CPE) accounting for the non-ideal capacitive behavior of the porous film electrode, and *W* is a Warburg element describing the low-frequency diffusion process. The fitted parameters, together with their uncertainties and the chi-squared (χ2) values, are summarized in Table S5 of the Supplementary Information. The fitted charge transfer resistance of the composite (726.6 Ω) is only about half that of pristine Zn_0.4_Cd_0.6_S (1522 Ω), quantitatively confirming that the heterojunction markedly reduces the interfacial charge transfer resistance, which is consistent with the PL and transient photocurrent results [42,73,77].

### 3.8. Proposed Photocatalytic Mechanism

**Figure 10:**
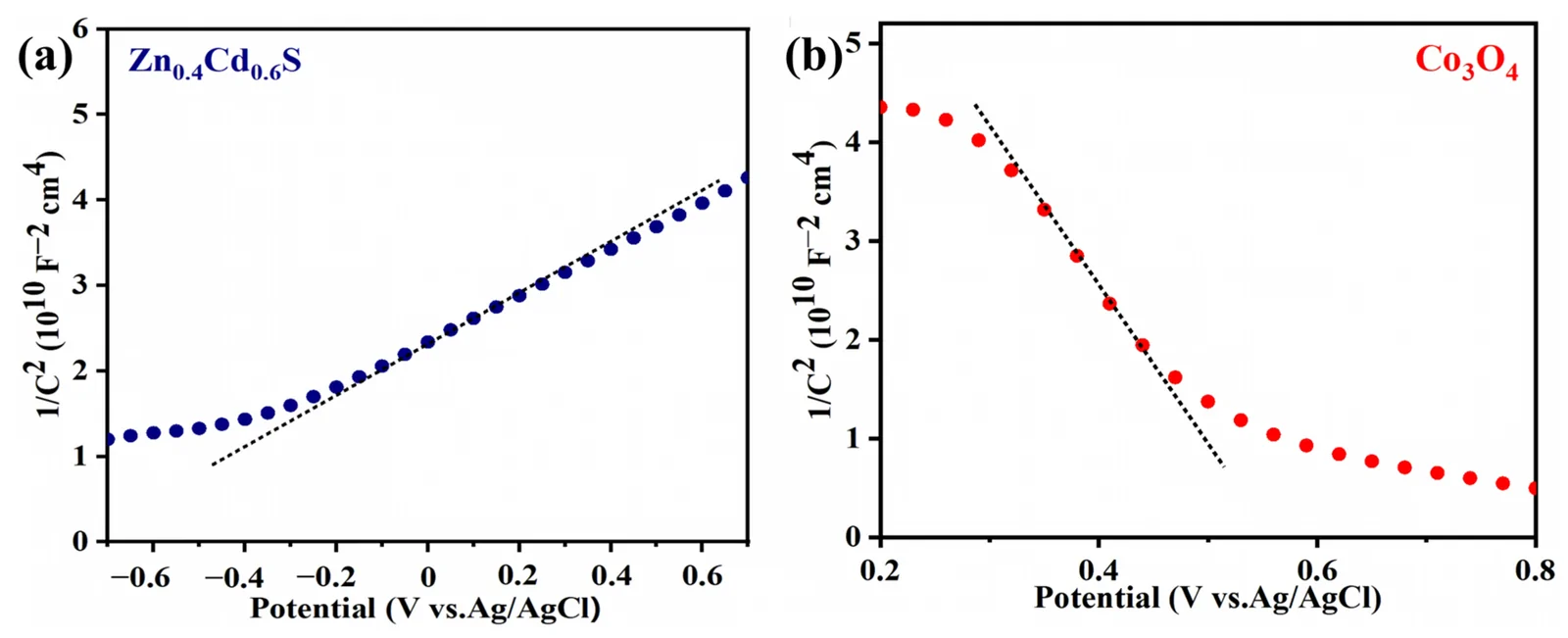
Mott–Schottky plots of Zn_0.4_Cd_0.6_S (**a**) and Co_3_O_4_ (**b**).

To determine the semiconductor types of pristine Co_3_O_4_ and Zn_0.4_Cd_0.6_S, Mott–Schottky measurements were carried out; the obtained curves depicted in Figure 10 show that Co_3_O_4_ exhibits a negative slope in the Mott–Schottky plot, signifying it as a p-type semiconductor, while Zn_0.4_Cd_0.6_S shows a positive slope, demonstrating its n-type semiconductor property. Therefore, the composite obtained by loading Co_3_O_4_ onto the Zn_0.4_Cd_0.6_S surface is identified as a p–n heterojunction [37].

**Figure 11:**
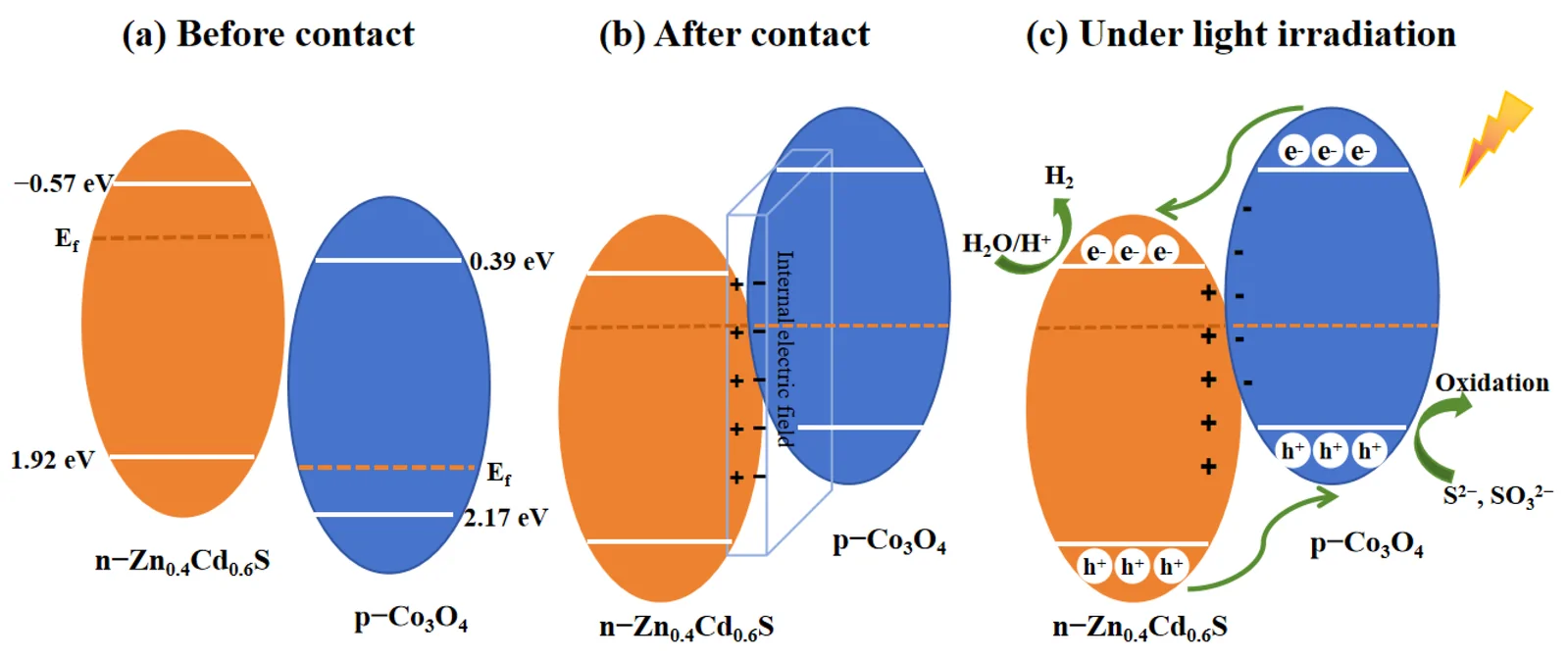
(**a**) Band structures of Co_3_O_4_ and Zn_0.4_Cd_0.6_S before contact; (**b**) formation of built-in electric field upon contact; (**c**) photoinduced charge separation and photocatalytic H_2_ evolution mechanism of the composite.

The photocatalytic mechanism of Co_3_O_4_/Zn_0.4_Cd_0.6_S is discussed as follows, along with the recombination process of p- and n-type semiconductors [37,83,90,92,93]. The band structure alignment and proposed mechanism are displayed in Figure 11. Based on prior calculations, the *E*_VB_ and *E*_CB_ of Co_3_O_4_ are 2.17 and 0.39 eV, and the *E*_VB_ and *E*_CB_ of Zn_0.4_Cd_0.6_S are 1.92 and −0.57 eV. The *E*_VB_ of Co_3_O_4_ is more positive than that of Zn_0.4_Cd_0.6_S, and the *E*_CB_ of Zn_0.4_Cd_0.6_S is more negative than that of Co_3_O_4_ before they come into contact (Figure 11a). It was hypothesized that the integration of Co_3_O_4_ and Zn_0.4_Cd_0.6_S could construct a type-II heterojunction. Under such a scenario, photoexcited electrons originating from Zn_0.4_Cd_0.6_S would migrate to Co_3_O_4_, while photogenerated holes in the valence band of Co_3_O_4_ would shift to Zn_0.4_Cd_0.6_S. Nevertheless, electrons accumulating in the conduction band of Co_3_O_4_ failed to drive water reduction to produce H_2_. This stems from the fact that the conduction band potential of Co_3_O_4_ is more positive than the the H^+^/H_2_ redox level [37]. Actually, the synthesized composite delivers greatly improved photocatalytic activity when compared to pristine Zn_0.4_Cd_0.6_S and pure Co_3_O_4_. Accordingly, the boosted photoactivity can be ascribed to the fabrication of a p–n heterojunction. When p-type Co_3_O_4_ comes into contact with n-type Zn_0.4_Cd_0.6_S, the Fermi level of Co_3_O_4_ rises, whereas that of Zn_0.4_Cd_0.6_S declines until their Fermi levels reach equilibrium. Electrons near the p–n interface transfer from n-type Zn_0.4_Cd_0.6_S to the p-type Co_3_O_4_ region. Meanwhile, holes migrate from p-type Co_3_O_4_ to n-type Zn_0.4_Cd_0.6_S. These accumulated charge species build up a space-charge region and further establish a built-in electric field (Figure 11b). Upon visible-light irradiation, driven by this built-in electrostatic field, photogenerated electrons in the CB of p-type Co_3_O_4_ transfer to the CB of n-type Zn_0.4_Cd_0.6_S and participate in the H_2_ evolution reaction. In contrast, photogenerated holes in the VB of n-type Zn_0.4_Cd_0.6_S migrate to the VB of p-type Co_3_O_4_, where they are consumed by sacrificial reagents (Figure 11c). The p–n heterojunction is well constructed as verified by Mott–Schottky measurements, enabling efficient separation of photogenerated charge carriers. This charge separation mechanism offers dual key advantages: on the one hand, the accumulated electrons on the Zn_0.4_Cd_0.6_S surface efficiently reduce H^+^ to H_2_; on the other hand, the holes concentrated on Co_3_O_4_ are effectively scavenged by sacrificial reagents. The established p–n heterojunction not only significantly enhances the charge separation efficiency but also suppresses the photocorrosion of the composite, resulting in synergistic improvement in photocatalytic performance and durability toward the H_2_ evolution from water splitting.

## 4. Conclusions

In summary, this work successfully developed a noble-metal-free Co_3_O_4_/Zn_0.4_Cd_0.6_S p–n heterojunction photocatalyst through a facile solvothermal approach. The optimized composite containing 4 wt% Co_3_O_4_ demonstrates exceptional photocatalytic performance with an outstanding hydrogen production rate reaching 56.63 mmol g^−1^ h^−1^, which is a 6.8-fold enhancement over pristine Zn_0.4_Cd_0.6_S and superior to many reported analogous photocatalysts. In this study, the effects of the Zn/Cd stoichiometric ratio and Co_3_O_4_ loading on photocatalytic activity were systematically explored to uncover the composition-dependent photocatalytic behavior. Systematic characterization reveals that the dramatically improved activity and stability originate from the well-constructed p–n heterojunction, which enables efficient spatial charge separation and transport while suppressing electron–hole recombination. Extending beyond the development of a high-efficiency, cost-effective photocatalyst, this study provides valuable guidelines for the rational design of heterojunction-based photocatalytic systems aimed at high-efficiency solar-to-chemical energy conversion.

## Figures and Tables

**Table 1:** The mass percentage of Co_3_O_4_ determined using ICP-OES analysis.

Composite Photocatalyst	The Co_3_O_4_ Content Determined Using ICP-OES Analysis (wt%)
1 wt%-Co_3_O_4_/Zn_0.4_Cd_0.6_S	0.99
2 wt%-Co_3_O_4_/Zn_0.4_Cd_0.6_S	2.02
3 wt%-Co_3_O_4_/Zn_0.4_Cd_0.6_S	3.03
4 wt%-Co_3_O_4_/Zn_0.4_Cd_0.6_S	4.03
5 wt%-Co_3_O_4_/Zn_0.4_Cd_0.6_S	4.99
6 wt%-Co_3_O_4_/Zn_0.4_Cd_0.6_S	5.96
7 wt%-Co_3_O_4_/Zn_0.4_Cd_0.6_S	6.98

## Data Availability

The original contributions presented in this study are included in the article/Supplementary Materials. Further inquiries can be directed to the corresponding author.

## Supplementary Materials

The following supporting information can be downloaded at: https://www.mdpi.com/article/10.3390/nano16211397/s1, Figure S1: XRD patterns of Zn_x_Cd_1−x_S (0 ≤ x < 1); Figure S2: Photocatalytic H_2_ evolution rate of Zn_x_Cd_1−x_S (0 ≤ x < 1) under visible-light irradiation; Figure S3: TEM images of bare Zn_0.4_Cd_0.6_S before (a) and after (b) the recycling experiments; Table S1: The molar ratio of Zn^2+^/Cd^2+^ in Zn_x_Cd_1−x_S (0 ≤ x ≤ 1); Table S2: The corresponding mean values, standard deviations (SD) and relative standard deviation (RSD) values of the photocatalytic H_2_ evolution rate of the samples; Table S3: Statistical summary of photocatalytic H_2_ evolution rates and Welch’s *t*-test results of Zn_0.4_Cd_0.6_S and 4 wt%-Co_3_O_4_/Zn_0.4_Cd_0.6_S; Table S4: Comparison of photocatalytic H_2_ production rates between Co_3_O_4_/Zn_0.4_Cd_0.6_S and analogous composites; Table S5: Fitted EIS parameters of Zn_0.4_Cd_0.6_S and Co_3_O_4_/Zn_0.4_Cd_0.6_S obtained with the equivalent circuit *R*_s_(*Q*(*R*_ct_*W*)).

## Abbreviations

The following abbreviations are used in this manuscript:

CB	Conduction band
VB	Valence band
SD	Standard deviations
RSD	Relative standard deviation
*E*_CB_	Conduction band potential
*E*_V__B_	Valence band potential
