# Supplementary material for: Efficient p–n Heterojunction Construction in Co3O4/Zn0.4Cd0.6S Composite for Boosted Visible-Light Photocatalytic H2 Production
Source: Nanomaterials (Basel). 2026 Oct 31;16(21):1397. doi: 10.3390/nano16211397 (PMC13765460; doi:10.3390/nano16211397)
Supplement: Supplementary file 1 [file nanomaterials-16-01397-s001.zip › nanomaterials-4550273-supplementary.pdf]

Supplementary Information

Efficient p–n heterojunction construction in $Co_3O_4/Zn_{0.4}Cd_{0.6}S$ composite for boosted visible-light photocatalytic H_2 production

Yanzhu Ye[1,2], Zhongshui Li[2], Alian Wang[1], Shen Lin[2,*]

[1] Training Management Division, Fujian Institute of Education, Fuzhou 350001, China; E-Mail: yanzhuye@126.com (Y.Y.); wal0922@163.com (A.W.)

[2] College of Chemistry and Materials Science, Fujian Normal University, Fuzhou 350007, China; E-Mail: zsli@fjnu.edu.cn (Z.L.)

* Correspondence: shenlin@fjnu.edu.cn

1. Experimental

1.1. Reagents and chemicals

Zinc nitrate hexahydrate ($Zn(NO_3)_2·6H_2O$), sodium sulfide nonahydrate ($Na_2S·9H_2O$), cadmium acetate dihydrate ($Cd(CH_3COO)_2·2H_2O$), ethylenediamine ($H_2NCH_2CH_2NH_2$), aqueous ammonia ($NH_3·H_2O$), cobalt nitrate hexahydrate ($Co(NO_3)_2·6H_2O$) and sodium sulfite (Na_2SO_3) were obtained from Xilong Scientific Co., Ltd. (Shantou, China). Argon (Ar) was acquired from Huaxinda Gas Co., Ltd. (Fuzhou, China). All reagents were used as received without further purification.

1.2. Characterization

XRD patterns of the samples were recorded utilizing a D8 ADVANCE diffractometer (Bruker Co., Karlsruhe, Germany). Elemental concentrations were measured by ICP-OES on an Agilent 720 instrument (Agilent Technologies Inc., USA). XPS analysis was carried out on an Al Kα-equipped ESCALAB 250Xi spectrometer (Thermo Fisher Scientific, USA). Optical absorption properties were evaluated from UV–Vis diffusive reflectance spectra acquired on a UV2700 spectrophotometer (Shimadzu, Japan). PL and TRPL measurements were conducted using an Edinburgh FLS980 spectrofluorometer (Edinburgh Instruments Ltd., UK). Microstructural analysis was conducted via TEM/HRTEM imaging using a Talos F200X microscope (Thermo Scientific, USA). A Micromeritics ASAP-2460 surface area analyzer (Micromeritics Instrument Corp., USA) was employed to determine the BET specific surface area of the products via N_2 adsorption. A CHI 760 electrochemical workstation

(Shanghai Chenhua Co., China) with a standard three-electrode cell was empolyed to perform the electrochemical and photoelectrochemical measurements. To prepare the working electrode, 5 mg of the sample was dispersed in a mixture of 2 mL ethanol and 20 μL of 5% Nafion solution under ultrasonic treatment for 30 minutes, followed by drop-casting onto FTO glass and vacuum drying at 120 °C for 6 h. Measurements were conducted in 0.5 mol/L Na_2SO_4 solution using Pt counter electrode paired with Ag/AgCl reference.

1.3 Photocatalytic activity test

The hydrogen evolution activity of the synthesized photocatalysts was evaluated using a CEL-SPH2N online system (Beijing MerryChange Technology Co., Ltd., Beijing, China) with 100 mL quartz reactor. 25 mg catalyst was ultrasonically dispersed in a 50 mL aqueous phase comprising 0.5 M Na_2S and Na_2SO_3. Prior to illumination, the system underwent 30-minute vacuum degassing. Visible light simulation was achieved using 420 nm filtered light from a 300 W Xe lamp. Hydrogen production was quantified hourly via automated gas chromatography (GC-7920) with high-purity argon as the carrier gas. Using the following formula, AQE values were obtained under identical measurement conditions:

$$AQE=\frac{2\times\text{the number of evolved } H_2 \text{ molecules}}{\text{the number of incident photons}}\times 100\%$$

1.4 Photoelectrochemical measurement

Transient photocurrent and electrochemical impedance spectroscopy (EIS) measurements were carried out in a three-electrode single-compartment electrolytic cell using a CHI 760 electrochemical workstation. For EIS tests, a rotating ring-disk electrode served as the working electrode, Ag/AgCl as the reference electrode, and a Pt wire as the counter electrode. In transient photocurrent measurements, FTO conductive glass was used as the working electrode, while Ag/AgCl and platinum wire acted as the reference and counter electrodes, respectively. All three electrodes were assembled in an electrolytic cell equipped with a quartz window. A designated electrolyte was adopted for EIS characterization, and 0.5 M Na_2SO_4 aqueous solution was used as the electrolyte for transient photocurrent tests. A 150 W xenon lamp fitted with a 420 nm cut-off filter was employed as the light source. All measured potentials were converted to the reversible hydrogen electrode (RHE) scale according to the equation:

$$RHE = E_{Ag/AgCl} + 0.059\ pH + 0.2224V - 0.059\lg a\ (Cl^-)$$

2. Results and Discussion

2.1 Composition and crystal phase structure analysis

Figure S1 illustrates the XRD patterns of the prepared $Zn_xCd_{1-x}S$ ($0 \leq x < 1$), indicating a hexagonal crystal system [57]. As shown in the figure, when x = 0, phase identification confirms the characteristic peaks at: 24.9° (100), 26.5° (002), 28.2° (101), 36.7° (102), 43.8° (110), 47.9° (103) and 51.9° (112), matching standard CdS diffraction data (JCPDS 41-1049) [17]. With increasing x values, the diffraction peaks of $Zn_xCd_{1-x}S$ ($0 < x < 1$) gradually shift toward higher diffraction angles, transitioning from hexagonal CdS to hexagonal ZnS. The reason for this phenomenon primarily lies in the smaller ionic radius of Zn^{2+} relative to that of Cd^{2+} [76]. As x increases, the smaller Zn^{2+} (0.74 Å) progressively replace the larger Cd^{2+} (0.97 Å), leading to a gradual reduction in the unit cell dimensions of $Zn_xCd_{1-x}S$ ($0 \leq x < 1$). Consequently, the diffraction peaks shift toward higher angles. The XRD analysis confirms that $Zn_xCd_{1-x}S$ ($0 < x < 1$) exists in the form of a solid solution rather than a simple mixture of CdS and ZnS [59, 70].

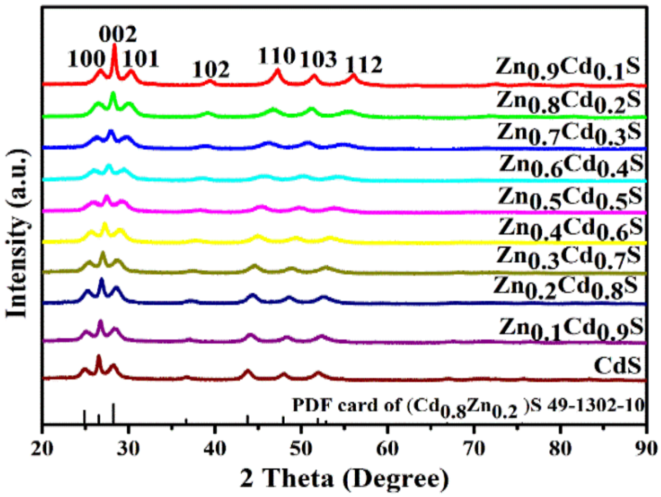

Figure S1. XRD patterns of $Zn_xCd_{1-x}S$ ($0 \leq x < 1$).

ICP-OES measurements were performed to determine the actual Zn/Cd molar ratios in $Zn_xCd_{1-x}S$ ($0 \leq x \leq 1$) solid solutions (Table S1). The experimental results indicate that the determined Zn^{2+}/Cd^{2+} molar ratio in the reaction precursors closely matches those obtained from ICP-OES analysis. For simplicity in notation, $Zn_xCd_{1-x}S$ ($0 \leq x \leq 1$) samples were named according to the molar ratios of Zn^{2+} to Cd^{2+} in the initial reaction mixtures.

Table S1. The molar ratio of Zn^{2+}/Cd^{2+} in $Zn_xCd_{1-x}S$ ($0 \leq x \leq 1$).

**$Zn_xCd_{1-x}S$（$0 \leq x \leq 1$）**	**ICP(Zn^{2+}: Cd^{2+})**
CdS	0 : 1[a]
$Zn_{0.1}Cd_{0.9}S$	0.099 : 0.901
$Zn_{0.2}Cd_{0.8}S$	0.207 : 0.793
$Zn_{0.3}Cd_{0.7}S$	0.297: 0.703

$Zn_{0.4}Cd_{0.6}S$	0.396: 0.604
$Zn_{0.5}Cd_{0.5}S$	0.502 : 0.498
$Zn_{0.6}Cd_{0.4}S$	0.594 : 0.406
$Zn_{0.7}Cd_{0.3}S$	0.693 : 0.307
$Zn_{0.8}Cd_{0.2}S$	0.810 : 0.190
$Zn_{0.9}Cd_{0.1}S$	0.909 : 0.091
ZnS	1 : 0[b]

a: single-component CdS control substance, b: single-component ZnS control substance.

2.2 Photocatalytic H_2 production

The photocatalytic hydrogen evolution activity of $Zn_xCd_{1-x}S$ ($0 \leq x \leq 1$) was evaluated under simulated solar light irradiation ($\lambda \geq 420$ nm) using 0.5 M Na_2S/Na_2SO_3 aqueous solution as the sacrificial agent. Control experiments confirmed that no H_2 was observed in either the light-irradiated system lacking a photocatalyst or the catalyst-containing system without light exposure.

Figure S2 presents the average hydrogen production rates of $Zn_xCd_{1-x}S$ ($0 \leq x < 1$) after 2 hours of irradiation. The pure CdS sample ($x = 0$) exhibited relatively weak photocatalytic activity with a hydrogen evolution rate of merely 0.73 mmol g^{-1} h^{-1}. Notably, the catalytic activity of $Zn_xCd_{1-x}S$ solid solutions enhanced significantly with increasing x values, reaching maximum performance at $x = 0.4$. $Zn_{0.4}Cd_{0.6}S$ displayed the most outstanding photocatalytic activity, yielding an optimal hydrogen production rate of 8.36 mmol g^{-1} h^{-1}, which represents an 11.5-fold enhancement compared to pure CdS.

However, further increase in x value beyond 0.4 leds to a gradual decline in photocatalytic activity. The $Zn_{0.9}Cd_{0.1}S$ sample ($x = 0.9$) showed a substantially reduced hydrogen evolution rate of 0.81 mmol g^{-1} h^{-1}, while pure ZnS ($x = 1$) exhibited no detectable hydrogen production. As evidenced by the photocatalytic testing, the Zn/Cd stoichiometric ratio serves as a critical tuning parameter for optimizing the photocatalytic performance of $Zn_xCd_{1-x}S$ solid solutions.

The superior activity of $Zn_{0.4}Cd_{0.6}S$ is likely attributed to its optimal band structure, which facilitates efficient separation and migration of photogenerated electrons and holes. Based on these findings, $Zn_{0.4}Cd_{0.6}S$ solid solution with the optimal composition was selected for subsequent investigations.

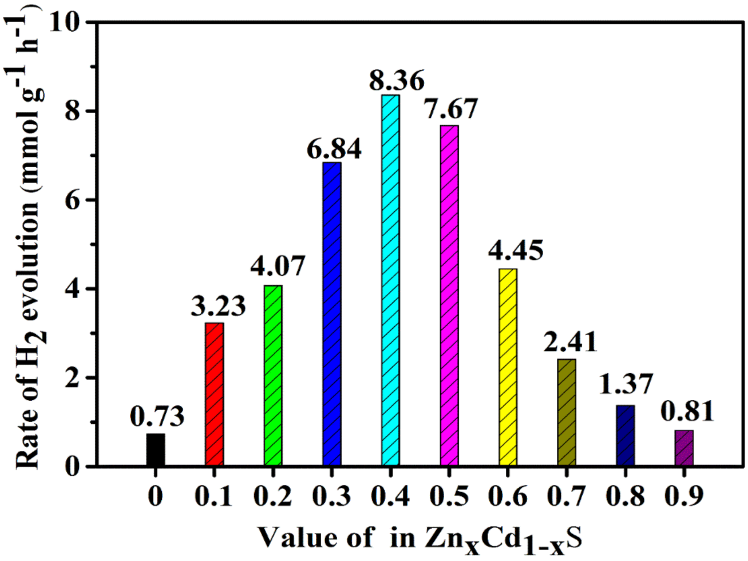

Figure S2. Photocatalytic H_2 evolution rate of $Zn_xCd_{1-x}S$ ($0 \leq x < 1$) under visible light irradiation.

The photocatalytic H_2 evolution rates are expressed as mean values obtained from three independent replicates. The standard deviations (SD) and relative standard deviation (RSD) values are summarized in Table S2. The RSD values of photocatalytic H_2 evolution rates for the as-synthesized products are 1.53%, confirming good measurement reproducibility.

Table S2. The corresponding mean values, standard deviations (SD) and relative standard deviation (RSD) values of the photocatalytic H_2 evolution rate of the samples.

Sample	Mean	SD	RSD (%)
$Zn_{0.4}Cd_{0.6}S$	8.36	0.43	5.18
1 wt%-$Co_3O_4/Zn_{0.4}Cd_{0.6}S$	23.75	1.00	4.21
2 wt%-$Co_3O_4/Zn_{0.4}Cd_{0.6}S$	41.72	1.22	2.93
3 wt%-$Co_3O_4/Zn_{0.4}Cd_{0.6}S$	49.92	1.04	2.09
4 wt%-$Co_3O_4/Zn_{0.4}Cd_{0.6}S$	56.63	0.87	1.53
5 wt%-$Co_3O_4/Zn_{0.4}Cd_{0.6}S$	47.08	1.38	2.94
6 wt%-$Co_3O_4/Zn_{0.4}Cd_{0.6}S$	41.15	0.59	1.43
7 wt%-$Co_3O_4/Zn_{0.4}Cd_{0.6}S$	38.65	1.46	3.79
1 wt%-$Pt/Zn_{0.4}Cd_{0.6}S$	19.72	0.87	4.43

A two-tailed Welch's two-sample t-test was carried out to compare the photocatalytic hydrogen evolution rates of bare $Zn_{0.4}Cd_{0.6}S$ and the $Co_3O_4/Zn_{0.4}Cd_{0.6}S$ composite. The statistical summary of photocatalytic H_2 evolution rates and Welch's t-test results is provided in Table S3. The calculated P

value is 4.19 × 10-6 ($P < 0.05$), confirming that the observed differences in photocatalytic H_2 evolution activity between the samples are statistically significant.

Table S3. Statistical summary of photocatalytic H_2 evolution rates and Welch's t-test results of $Zn_{0.4}Cd_{0.6}S$ and 4 wt%-$Co_3O_4/Zn_{0.4}Cd_{0.6}S$.

Sample	N	Mean	SD
$Zn_{0.4}Cd_{0.6}S$	3	8.36	0.43
4 wt%-$Co_3O_4/Zn_{0.4}Cd_{0.6}S$	3	56.63	0.87

Welch's two-tailed t-test: t = -86.39 , df = 2.94 , P = 4.19×10^{-6}. $P < 0.05$, the difference is statistically significant.

Table S4. Comparison of photocatalytic H_2 production rates between $Co_3O_4/Zn_{0.4}Cd_{0.6}S$ and analogous composites.

Photocatalyst	Optimal H_2 Evolution Rate (mmol g^{-1} h^{-1})	Ref.
$Co_3O_4/Zn_{0.4}Cd_{0.6}S$	56.63 mmol g^{-1} h^{-1}	This work
$Zn_{0.4}Cd_{0.6}S$	8.36 mmol g^{-1} h^{-1}	This work
1.0 wt%–$Pt/Zn_{0.4}Cd_{0.6}S$	19.72 mmol g^{-1} h^{-1}	This work
$Mn_{0.25}Cd_{0.75}S/Co_3O_4$	25.71 mmol g^{-1} h^{-1}	[37]
$C/MoS_2@ZnIn_2S_4/Co_3O_4$	6.7 mmol g^{-1} h^{-1}	[41]
$Mn_{0.2}Cd_{0.8}S/MoS_2/Co_3O_4$	16.45 mmol g^{-1} h^{-1}	[42]
$Ni(OH)_2$ / $Zn_{0.8}Cd_{0.2}S$	7.16 mmol g^{-1} h^{-1}	[43]
$CdS@MoS_2–Co_3O_4$	27.8 mmol g^{-1} h^{-1}	[44]
$Co_3O_4@ZnIn_2S_4$	4.515 mmol g^{-1} h^{-1}	[45]
$Zn_{0.5}Cd_{0.5}S/CoP$	12.1758 mmol g^{-1} h^{-1}	[46]
$Zn–CoP/ZnIn_2S_4$	24.9 mmol g^{-1} h^{-1}	[47]
$Ag_2S/Zn_{0.6}Cd_{0.4}S$	18.77 mmol g^{-1} h^{-1}	[48]
$PtPd/Zn_{0.5}Cd_{0.5}S$	9.689 mmol g^{-1} h^{-1}	[49]
$Pt@Co_3O_4/TiO_2$	19.2 mmol g^{-1} h^{-1}	[50]
C@CdS–HS/Pt	20.9 mmol g^{-1} h^{-1}	[51]
Co_3O_4–9%/ZnCdS	2042 μmol g^{-1} h^{-1}	[52]
$Co_3O_4/CdS/Cd_{0.805}Zn_{0.195}S$	5231.44 μmol g^{-1} h^{-1}	[53]
$Co_3O_4/Cd_{0.8}Zn_{0.2}S$	6816.52 μmol μmol g^{-1} h^{-1}	[54]

CoP/ZnCdS/Co_3O_4	24.2 mmol g^{-1} h^{-1}	[55]
$Zn_{0.5}Cd_{0.5}S$–Co_3O_4db–20 %	33885 μmol μmol g^{-1} h^{-1}	[56]
$Ni_2P/Cd_{0.5}Zn_{0.5}S/Co_3O_4$	39.66 mmol g^{-1} h^{-1}	[57]
C@$Zn_xCd_{1-x}S/Co_3O_4$ (30%)	1405.1 μmol μmol g^{-1} h^{-1}	[58]

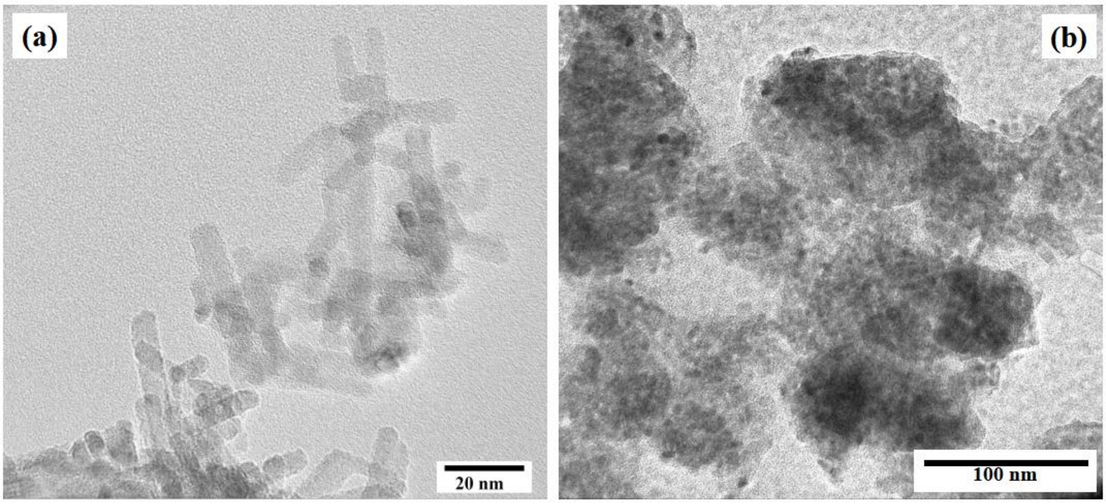

Figure S3. TEM images of bare $Zn_{0.4}Cd_{0.6}S$ before (a) and after (b) the recycling experiments.

Table S5. Fitted EIS parameters of $Zn_{0.4}Cd_{0.6}S$ and $Co_3O_4/Zn_{0.4}Cd_{0.6}S$ obtained with the equivalent circuit $R_s(Q(R_{ct}W))$.

**Parameter**	**$Zn_{0.4}Cd_{0.6}S$**	**$Co_3O_4/Zn_{0.4}Cd_{0.6}S$**
*R*s (Ω)	69.09 ± 1.14	55.31 ± 0.85
CPE-T (S·sn)	(1.11 ± 0.08) × 10-5	(1.63 ± 0.13) × 10-5
CPE-P (n)	0.930 ± 0.012	0.916 ± 0.013
*R*ct (Ω)	1522 ± 28	726.6 ± 12.8
χ2	6.77 × 10-3	5.73 × 10-3

References

References for the Supplementary Information are provided in the main manuscript.
